# Spatiotemporal variation of small hive beetle infestation levels in honeybee host colonies

**DOI:** 10.1007/s13592-025-01206-8

**Published:** 2025-09-04

**Authors:** Aura Palonen, Anna Papach, Michael N. K. Muturi, Érica Weinstein Teixeira, Geoffrey R. Williams, Rachel Jacobsen, Jay D. Evans, Francisco J. Posada-Florez, Christian W. W. Pirk, H. Michael G. Lattorff, Akinwande K. Lawrence, Murele O. Oluseyi, Robert Spooner-Hart, Clarissa M. House, Giovanni Federico, Giovanni Formato, Peter Neumann

**Affiliations:** 1 Institute of Bee Health, Vetsuisse Faculty, University of Bern, Bern, Switzerland; 2 International Centre of Insect Physiology and Ecology (icipe), Nairobi, Kenya; 3 Instituto Biológico, Agência Paulista de Tecnologia dos Agronegócios/SAA-SP, Pindamonhangaba SP, Brazil; 4 Department of Entomology & Plant Pathology, Auburn University, Auburn, AL USA; 5 U.S. Department of Agriculture -Agricultural Research Service (USDA-ARS) Bee Research Laboratory, Beltsville, USA MD; 6 Social Insects Research Group, Department of Zoology and Entomology, University of Pretoria, Private Bag X20, Pretoria, Hatfield South Africa; 7 School of Life Sciences, University of KwaZulu-Natal, Westville Campus, Durban, South Africa; 8 Department of Biology, Federal University of Technology, Akure, Nigeria; 9 Hawkesbury Institute for the Environment, Western Sydney University, Penrith, NSW Australia; 10 School of Science, Western Sydney University, Richmond, NSW Australia; 11 Istituto Zooproﬁlattico Sperimentale del Mezzogiorno, Laboratorio di Patologia Apistica, Reggio Calabria, Italy; 12 Istituto Zooprofilattico Sperimentale del Lazio e della Toscana “M. Aleandri”, Rome, Italy

**Keywords:** *Aethina tumida*, *Apis mellifera*, Host, Parasite, Invasive species, Colony loss

## Abstract

High infestation levels of small hive beetle (SHB), *Aethina tumida*, can cause more damage to honeybee, *Apis mellifera*, host colonies. However, the spatiotemporal variation of SHB infestations is poorly understood. Here, we show that SHB infestations can be equally high in native and invasive ranges, suggesting that differences between host populations are the key criterion for damage. The data reveal that spatial variation within locations was not correlated with migratory beekeeping, SHB management strategies, nor the number of colonies at an apiary. Despite no annual changes in SHB infestations, the data confirm seasonal variation in infestations in two locations probably due to environmental factors affecting SHB. Infestations are lower in Italy than elsewhere, possibly due to strongly implemented management strategies. It is apparent that our understanding of varying SHB infestations is still limited. This suggests that further efforts are required to elucidate our knowledge of this important host-parasite system.

**Supplementary Information:**

The online version contains supplementary material available at 10.1007/s13592-025-01206-8.

## Introduction

Fluctuations in parasite prevalence and abundance are often driven by a complex web of host-related factors, such as demographic traits including host density and abundance, and environmental variables, such as seasonal and spatial variations in humidity and temperature (Sweeny et al. 2021; Majewska et al. 2022; Byers et al. 2008; Froeschke et al. 2010; Sánchez-Hernández 2017). Monitoring parasite abundance is essential to predict their impacts on hosts, since negative fitness consequences on hosts tend to increase with parasite load (Hudson et al. 2002). Understanding the dynamics of spatiotemporal variation in parasite-host systems is especially crucial in the context of invasive parasites, as it assists in developing management strategies (Telfer and Bown 2012).

The small hive beetle (SHB), *Aethina tumida* Murray (Coleoptera, Nitidulidae), is a parasite of Western honeybee (*Apis mellifera*) colonies endemic to sub-Saharan Africa (Lundie 1940), and it has spread to all continents except Antarctica (Neumann et al. 2016; Papach et al. 2023). Adult SHBs reproduce in the colony, and the larvae feed on pollen, honey, and bee brood before leaving the colony to pupate in the soil (Lundie 1940; Neumann et al. 2016). In its endemic range in sub-Saharan Africa, SHB commonly infests local honeybee colonies (Hepburn and Radloff 1998) and rarely causes severe damage to its hosts (Lundie 1940; Pirk and Yusuf 2015). However, in the invasive ranges, SHB can cause considerable damage, especially to European (-derived) honeybee colonies in the USA and Australia (Neumann and Elzen 2004; Neumann et al. 2016; Spooner-Hart et al. 2017). This damage is commonly associated with SHB mass reproduction, where up to thousands of larvae can cause the entire colony to collapse by feeding on bee brood, honey, and pollen (Hepburn and Radloff 1998; Neumann and Elzen 2004).

The reported differences in damage caused by SHB have been linked to quantitative differences in a range of behaviours between African and European (-derived) honeybee hosts (Elzen et al. 2001; Neumann and Elzen. 2004; Neumann et al. 2018). Higher regional SHB infestation levels in more heavily damaged areas (Spiewok et al. 2007) may be driven by enemy release (Neumann et al. 2016) as well as environmental conditions, such as soil moisture and temperature (Cornelissen et al. 2019).

These environmental conditions mostly affect the development and survival of SHB larval and pupal stages outside of their host colonies, possibly limiting the number of annual generations (Ellis et al. 2004; Bernier et al. 2014; Cornelissen et al. 2019). In addition to spatial differences in temperatures, temporal changes also seem to play a role in SHB abundance: invasive SHB populations have been reported to fluctuate seasonally, with higher infestation levels observed during summer (Arbogast et al. 2010; de Guzman et al. 2010; Annand 2011). Since the lower threshold temperature for successful SHB pupation has been estimated to be around 10 °C (Bernier et al. 2014), it is likely that population growth slows down over cooler months and peaks during warmer temperatures (Cornelissen et al. 2019). Furthermore, while SHB can pupate under a wide range of conditions, success is lower in very dry or very wet substrates (Ellis et al. 2004; Bernier et al. 2014; Cornelissen et al. 2019, 2020). Indeed, the optimal humidity conditions created by the seasonal rainfall patterns most likely facilitated the spread of SHB in India (Kumaranag et al. 2025) and may also cause temporal variation in local SHB infestations. Reports of SHB infestation levels with respect to precipitation in the native range are conflicting, with higher infestation levels during rainy seasons in Kenya (Torto et al. 2010; Arbogast et al. 2012), but during dry seasons in Nigeria (Lawal and Banjo 2008). Since the observations from Kenya and Nigeria are so different, this suggests that factors other than seasonal changes in environmental conditions can also be important predictors of SHB infestation levels (Neumann et al. 2016).

SHB infestation levels also differ significantly within apiaries of the same region (Spiewok et al. 2007). Since proximity to honey houses has been shown to correlate with SHB infestations (Spiewok et al. 2007), it is likely that other beekeeping practices may also explain some of these variations by affecting both host density (the number of colonies in an apiary) and host susceptibility (Neumann et al. 2016). For example, very tight spacing between frames decreases the ability of a honeybee colony to defend its nest against SHB (Meikle et al. 2015). A higher number of colonies at an apiary may result in more olfactory cues for SHB (Bobadoye et al. 2018) and could attract more free-flying individual SHB (Cornelissen et al. 2024) or SHB swarms (Tribe 2000), boosting local infestation levels. Beekeepers may also reduce local SHB infestations by using management, e.g. traps (Neumann and Hoffmann 2008; Bernier et al. 2015; Neumann et al. 2016; Muturi et al. 2022). Further, even short-range movement may stress colonies, e.g. frames sticking to hive walls, thereby promoting SHB mass reproduction in the apiaries, where colonies were moved.

Although SHB infestation levels are known to vary temporally, specific changes have only been studied in one native population in the Republic of South Africa (RSA), where prevalence was higher in the winter of 2011 compared to the previous year (Strauss et al. 2013). Since data on annual variations in infestation levels could help predict both the effect on their hosts and the spread of the invasive populations, further research into annual changes in SHB infestation levels is warranted. Despite multiple recent SHB invasions (Papach et al. 2023; Hossain et al. 2024; Kumaranag et al. 2025), broad-scale monitoring of SHB infestation levels in its native and invasive ranges has not been undertaken since 2007 (Spiewok et al. 2007). In conclusion, the drivers of SHB infestations of host colonies are not well understood.

In this study, we estimated SHB infestation levels across spatial and temporal scales in multiple native and invasive populations and investigated the impacts of past migratory beekeeping, pest management practices, and the number of co-located colonies. We expect differences in SHB infestation levels among locations, with higher infestation levels in well-established invasive populations and lower infestation levels in native populations and newly established invasive populations (Spiewok et al. 2007). Furthermore, we expect a negative effect of SHB management on infestation levels, a positive effect of the number of colonies at an apiary on infestation levels, and a positive effect of movement of colonies into an apiary on local infestation levels. Additionally, we re-investigated temporal variation in SHB infestation levels over both years and seasons in invasive and native populations. We hypothesized higher infestation levels in warmer months and during/immediately after rainy seasons and no annual changes in local infestation levels in native and established invasive populations.

## Material and methods

### Sampling

Data were collected at each of the eight locations when SHB infestation levels were assumed to be high based on published literature or information from local beekeepers (Table I). In each location, three to four apiaries were chosen that were at least 2 km apart and known to be naturally infested with SHB. Within each apiary, four to ten honeybee, *Apis mellifera*, colonies were randomly selected and visually screened for SHB using modified standard methods (Spiewok et al. 2007; Neumann et al. 2013; Cornelissen and Neumann 2018). In brief, the lid, each frame, and bottom of the hive were thoroughly screened, and all SHB were collected with an aspirator without shaking bees off the frame. During the inspection of each frame, the lid of the hive was closed to prevent SHB from escaping. Inspected frames were placed in an empty hive box, which was closed with a lid. Afterwards, the frames were moved back to the original hive box, and the second hive box was screened in case any SHB were missed during the initial inspection. The inspection of one hive took approximately 20–60 min. SHB infestation levels were quantified as the number of SHB found in the colony. Additionally, we recorded the number of colonies per apiary at the time of data collection and past migratory history (if any colonies at the apiary were moved during the previous year) as well as any management practices conducted against SHB during the previous year.

**Table I:** Summary of sampling locations and timelines of estimating small hive beetle (SHB), *Aethina tumida*, infestation levels in honeybee, *Apis mellifera*, colonies (*N* = 321). Data collection sites are characterized by range type (invasive or native), location, area, and apiary ID. The table also reports the timing and number of colonies of the first and second data collection events. The data collection times in **bold** show the data that were included in the analysis of temporal variation in SHB infestation levels. All data except for the data collection times in **bold and underlined** were included in the analysis of spatial variance in SHB infestation levels. References for relevant literature of seasonal variation in SHB infestation levels are included in the final column, where available

Data collection sites				1^st^screening		2^nd^screening		**﻿Reference**
Range	Location	Area	Apiary	Time	N colonies	Time	N colonies	
Native	South Africa	Gauteng	RSA1	Feb 2023	10	–	–	Lundie 1940
			RSA2	**Nov 2022**	10	**Feb 2024**	10	
			RSA3	Apr 2023	10	–	–	
Native	Kenya	Nairobi City	KE1	**Jul- Aug 2021**	4	**Nov 2022**	5	Arbogast et al. 2010; Torto et al. 2010
			KE2	**Jul- Aug 2021**	4	**Nov 2022**	5	
			KE3	Jul- Aug 2021	4	–	–	
Native	Nigeria	Osun	NI1	Jul 2023	10	–	–	Akinwande and Neumann 2018
		Ondo	NI2	Jul 2023	10	–	–	
		Osun	NI3	Jul 2023	10	–	–	
Invasive	USA	Alabama	AL1	**Jul 2022**	9	**May 2023**	10	de Guzman et al. 2010
			AL2	Jul 2022	10	–	–	
			AL3	Jul 2022	10	–	–	
Invasive	USA	Maryland	MD1	May 2023	10	–	–	
			MD2	Jun 2023	10	–	–	
			MD3	Jun 2023	10	–	–	
Invasive	Brazil	Rio de Janeiro	BR1	**Apr 2022**	10	**Feb 2023**	10	
		Rio de Janeiro	BR2	**Apr 2022**	10	**Feb 2023**	10	
		Rio de Janeiro	BR2	**Apr 2022**	10	**Feb 2023**	10	
		Saõ Paulo	BR4	–	–	Feb 2023	10	
Invasive	Italy	Reggio Calabria	IT1	**Apr 2022**	10	**May 2023**	10	
			IT2	**Apr 2022**	10	**May 2023**	10	
			IT3	**May 2022**	10	**May 2023**	10	
Invasive	Australia	New South Wales	AUS1	Feb 2023	10	–	–	
			AUS2	Feb 2023	10	–	–	
			AUS3	Feb 2023	10	–	–	

### Statistical analyses

All data analyses were performed using R version 4.5.0 (R Core Team 2024). Figures were made with R package ggplot2 (Wickham 2016) and tables with R package flextable (Gohel and Skintzos 2024).

#### Spatial variation

Whenever SHB infestations were measured at the same apiaries at two timepoints, the data were tested for significant differences between these timepoints, and if none were found, both timepoints were included in the analysis of spatial variation in SHB infestations (Table I). In cases where the timepoints differed (Kenya and RSA, Table II), the timepoint with higher SHB infestation levels was included in the final dataset to limit possible temporal variation for this spatial comparison.

**Table II:** Temporal variation in small hive beetle (SHB), *Aethina tumida*, infestation levels of honeybee, *Apis mellifera*, colonies. Ranges, locations, areas, sample sizes of apiaries and colonies, times of 1 st and 2nd screenings, type of comparison (annual or seasonal), total numbers of SHBs found at 1 st and 2nd screenings, test statistics and *p*-values are shown. Significant *p*-values are shown in **bold**. While no significant annual differences in SHB infestation levels were found, differences between seasons were significant. SHB infestation levels were higher at the beginning of the dry season compared to the end and in summer compared to spring

Range	Location	Area	N apiaries	N colonies	1 st screening	2nd screening	Comparison	N SHB 1 st screening	N SHB 2nd screening	Test statistic	*p*
Invasive	USA	Alabama	1	19	Jul 2022	May 2023	Annual	295	283	*t* = 0.224	0.826
	Brazil	Rio de Janeiro	3	60	Apr 2022	Feb 2023	Annual	563	664	*W* = 361.5	0.268
	Italy	Calabria	3	60	Apr–May 2022	May 2023	Annual	86	85	*W* = 414.5	0.585
Endemic	Kenya	Nairobi	2	16	Jul–Aug 2021	Nov 2022	Seasonal (middle vs. end of dry season)	583	255	*W* = 65	**0.029**
	South Africa	Gauteng	1	20	Nov 2022	Feb 2024	Seasonal (spring vs. summer)	731	1514	*W* = 21	**0.031**

We used generalized linear mixed models (GLMM) to assess the effect of location, past colony movements, and colony management practices against SHB infestation levels. The data were fit to GLMMs with negative binomial error distribution and log link function using R package lme4 (Bates et al. 2015). First, a full model was fitted including number of SHB per hive as the response variable and location, number of colonies at apiary, past migratory history at apiary (yes/no), and past management history against SHB at apiary (yes/no) as predictor variables. Number of colonies at an apiary was standardized by subtracting the mean and dividing by standard deviation to improve model convergence. Apiary was added as a random effect in the model to estimate apiary-level variation by allowing the intercept to vary by apiary. The full model was then compared to a set of nested models, where each predictor was removed one at a time, using likelihood ratio tests. Predictors that did not significantly improve the model fit were removed. The significance of each predictor of the final model was then determined with likelihood ratio tests as described above. To test whether the random effect improved model fit, the final model including the random effect was compared to a model without. To ensure that the residuals of the final model met model assumptions, diagnostic testing of residuals was performed with R package DHARMa (Hartig and Lohse 2022). Since location was a significant predictor in the final model, differences in SHB infestation levels between the different locations predicted by the model were assessed with post hoc pairwise comparisons of the estimated marginal means (EMMs) using R package emmeans (Lenth et al. 2023).

#### Temporal variation

SHB infestation levels were compared between seasons in Kenya and RSA and between years in Italy, Brazil, and the USA (Table II). The data from each location were tested for normality using Shapiro–Wilk tests and for equal variances using *F*-tests. The data from Alabama, USA, met the assumptions of normality (*W* = 0.95, *p* = 0.361; *F* = 0.961, *p* = 0.951), while the data from Italy and Brazil did not (Italy, *W* = 0.565, *p* < 0.001; Brazil, *W* = 0.895, *p* < 0.001). Therefore, differences in the number of SHB in a hive between 2022 and 2023 were tested in Alabama, USA, with a *t*-test and in Italy and Brazil with Wilcoxon rank-sum tests. The data from RSA and Kenya did not meet the assumptions of normality (RSA spring, *W* = 0.841, *p* = 0.046; Kenya, *W* = 0.803, *p* = 0.002). Differences in the number of SHB in a hive between spring and summer in RSA and between the middle and the end of the dry season in Kenya were therefore tested using Wilcoxon rank-sum tests.

## Results

### Spatial variation

Results of the full and final models are presented in Table III. Median SHB infestation levels from all apiaries and locations are presented in Table SI. Location was a significant predictor of the number of SHB in a colony (Table III, *χ*^2^ = 36.4, *p* < 0.001). However, the variance of the random effect being larger than zero (Table III, variance = 0.347) and the variation in the random intercepts within locations (Fig S1) indicate that apiary-level differences also contributed to the variation in infestation levels.

**Table III:** Effects of the predictor variables on small hive beetle (SHB), *Aethina tumida*, infestation levels of honeybee, *Apis mellifera*, colonies. The results of two generalized linear mixed models are shown. The full model includes all candidate predictor variables, whereas the final model was refined to only significant predictor variables. For fixed effects, estimated coefficients and their standard errors along with the *z*-values and *p*-values are presented. The overall significance of the predictor variable location in the final model was determined by comparing the model with the variable to a model without the variable using likelihood ratio tests, and the associated *χ*^2^ test statistic and *p*-value are presented in the footnote. For random effects, the variance and standard deviation are presented. Location was the only predictor variable that had a significant effect on SHB infestation levels of honeybee colonies. Significant *p*-values are in **bold** (*β* ± s.e. = estimated coefficient ± standard error, Std. dev. = standard deviation)

Fixed effects	Full model			Final model		
	***β*** ± s.e	***z*-**value	*p*-value	***β*** ± s.e	***z***-value	*p****-value***
Intercept	3.946 ± 0.364	10.833	< 0.001	3.939 ± 0.369	10.685	** < 0.001**
Location: Brazil	− 0.565 ± 0.638	− 0.885	0.376	− 1.019 ± 0.482	− 2.114	**0.035**
Location: Italy	− 2.941 ± 0.989	− 2.974	0.003	− 3.429 ± 0.533	− 6.428	** < 0.001**
Location: Kenya	0.747 ± 0.616	1.213	0.225	0.339 ± 0.548	0.618	0.537
Location: Nigeria	− 0.248 ± 0.515	− 0.482	0.629	− 0.241 ± 0.521	− 0.462	0.644
Location: RSA	0.621 ± 0.503	1.235	0.217	0.623 ± 0.521	1.195	0.232
Location: USA Alabama	− 0.248 ± 0.743	− 0.334	0.739	− 0.845 ± 0.519	− 1.628	0.103
Location: USA Maryland	− 1.317 ± 0.565	− 2.33	0.02	− 1.349 ± 0.523	− 2.58	**0.01**
Colony movement	− 0.606 ± 0.478	− 1.268	0.205			
Management against SHB	− 0.101 ± 0.76	0.133	0.894			
Number of colonies	0.009 ± 0.146	0.061	0.951			
**Random effects**	**Variance**	**Std. dev**		**Variance**	**Std. dev**	
Intercept: apiary	0.32	0.565		0.346	0.59	

N observations: 301 colonies and 25 apiaries

Overall significance of the variable location in the final model: *Χ*^2^ = 36.39, *p*-value < 0.001

**Figure 1.**
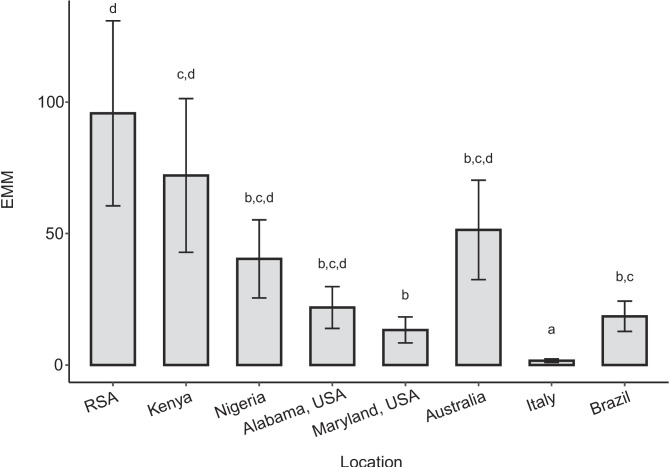
Spatial variation of small hive beetle (SHB), *Aethina tumida*, infestations of honeybee, *Apis mellifera*, host colonies across eight locations. The predicted number of SHBs in a colony is presented as estimated marginal means (EMM) and their standard errors (tops of the bars and whiskers) that were derived from a generalized linear mixed model. EMMs were back-transformed from the log scale to the original response scale by exponentiating. Bars sharing the same letter indicate groups that are not significantly different, based on Tukey-adjusted pairwise comparisons of the EMMs

The results from the pairwise comparisons of EMMs of the number of SHB in a honeybee colony across all locations are presented in Table IV. The estimated number of SHB per honeybee colony in Italy was significantly lower than in all other locations (Fig. 1). Additionally, the EMM of the number of SHB in a colony was significantly lower in Maryland compared to RSA and Kenya (Fig. 1) and in Brazil compared to RSA (Fig. 1).

**Table IV:** Differences in predicted numbers of small hive beetles (SHB), *Aethina tumida*, per honeybee, *Apis mellifera*, colony between locations. Pairwise comparisons of the estimated marginal means for each level of the variable location, derived from predictions of a generalized linear mixed model (GLMM), are shown. For each comparison, estimates and standard errors along with *z*-ratios and associated *p*-values are presented. Comparisons where the locations are significantly different are highlighted in **bold**. Note that the comparisons are on the log scale since the GLMM was fit with the log link function

Comparison	Estimate ± s.e	*z*-ratio	*p*-value
Australia—Brazil	1.019 ± 0.482	2.117	0.404
**Australia—Italy**	**3.428 ± 0.533**	**6.435**	** < 0.001**
Australia—Kenya	− 0.3389 ± 0.548	− 0.619	0.999
Australia—Nigeria	0.241 ± 0.521	0.463	0.999
Australia—RSA	− 0.623 ± 0.52	− 1.197	0.933
Australia—USA Alabama	0.854 ± 0.518	1.648	0.721
Australia—USA Maryland	1.349 ± 0.522	2.583	0.162
**Brazil—Italy**	**2.409 ± 0.495**	**4.865**	** < 0.001**
Brazil—Kenya	− 1.358 ± 0.511	− 2.660	0.135
Brazil—Nigeria	− 0.779 ± 0.481	− 1.617	0.74
**Brazil—RSA**	** − 1.642 ± 0.481**	** − 3.413**	**0.015**
Brazil—USA Alabama	− 0.166 ± 0.479	− 0.347	1
Brazil—USA Maryland	0.329 ± 0.56	0.682	0.998
**Italy—Kenya**	** − 3.767 ± 0.56**	** − 6.732**	** < 0.001**
**Italy—Nigeria**	** − 3.187 ± 0.533**	** − 5.979**	** < 0.001**
**Italy—RSA**	** − 4.051 ± 0.533**	** − 7.606**	** < 0.001**
**Italy—USA Alabama**	** − 2.575 ± 0.53**	** − 4.855**	** < 0.001**
**Italy—USA Maryland**	** − 2.079 ± 0.535**	** − 3.889**	**0.003**
Kenya—Nigeria	0.58 ± 0.548	1.059	0.965
Kenya—RSA	− 0.284 ± 0.547	− 0.518	0.999
Kenya—USA Alabama	1.192 ± 0.545	2.187	0.36
**Kenya—USA Maryland**	**1.688 ± 0.549**	**3.073**	**0.044**
Nigeria—RSA	− 0.863 ± 0.52	− 1.660	0.937
Nigeria—USA Alabama	0.613 ± 0.518	1.183	0.937
Nigeria—USA Maryland	1.108 ± 0.518	2.122	0.401
RSA—USA Alabama	1.476 ± 0.518	2.852	0.083
**RSA—USA Maryland**	**1.972 ± 0.522**	**3.778**	**0.004**
USA Alabama—USA Maryland	0.495 ± 0.520	0.953	0.981

Past migratory history, past management activities against SHB, and the number of colonies at an apiary did not significantly predict the number of SHB in a colony (Table III) so were removed from the final model to improve model fit.

### Temporal variation

**Figure 2.**
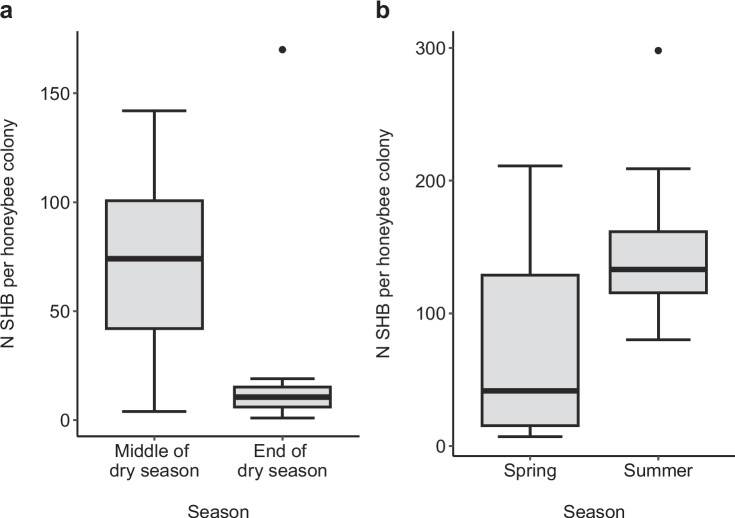
Seasonal variation in small hive beetle (SHB), *Aethina tumida*, infestations of honeybee, *Apis mellifera*, host colonies in two populations in the native range. The box plots show SHB counts per colony in **a** Kenya during the middle and the end of the dry season and **b** in RSA during spring and summer. Each box plot displays medians, 25th and 75th percentiles, and 10th and 90th percentiles, as well as individual data points falling outside these ranges. SHB counts per honeybee hive were significantly higher in the middle of the dry season than at the end in Kenya and in summer compared to spring in RSA (Wilcoxon rank-sum tests)

The results comparing numbers of SHB in a colony in different years and seasons are presented in Table II. Numbers of SHB in a colony did not significantly differ between the two years in any of the locations (Table III; Alabama, USA, *t* = 0.224, *p* = 0.826; Brazil, *W* = 361.5, *p* = 0.268; Italy, *W* = 414.5, *p* = 0.585). Median number of SHB in a colony was significantly higher during summer compared to spring (Fig. 2). Median number of SHB in a hive was significantly higher in the middle compared to the end of the dry season in Kenya (Fig. 2).

## Discussion

The data show for the first time that SHB infestation levels can be equally high in native and invasive ranges, suggesting that differences between the host populations are the key criterion for damage. Apiary-level differences contributed to the spatial variation in SHB infestation levels, but this variation was not correlated with past movement of colonies, past SHB management, or the total number of colonies in the apiary. While there were no annual changes, the data support previous reports of seasonal variation in SHB infestations (Torto et al. 2010; Arbogast et al. 2012; Neumann et al. 2016), i.e. higher infestation levels in summer than in spring in RSA, and in the middle of the dry season compared to the end in Kenya. Based on published reports (de Guzman et al. 2010), it seems as if seasonal changes are not restricted to the native range in Africa.

SHB infestations of honeybee colonies were similar to or higher, rather than lower in the native range compared to well-established invasive populations in Australia and southeast USA. There were no significant differences in SHB infestation levels among RSA, Kenya, Nigeria, Australia, and Alabama, and infestation levels in Brazil were only significantly lower than RSA. These findings show that higher SHB infestation levels are not confined to areas with higher damage to honeybee hosts, as beetle-induced colony losses are rare in the native range of SHB (Hepburn and Radloff 1998; Neumann et al. 2016) despite the observed high infestation levels. Therefore, the number of adult SHB in a given colony *per se* appears not to be a reliable predictive marker for the damage to the host. Indeed, high infestation levels alone do not necessarily lead to colony collapses, as local honeybee populations in the native range of SHB can withstand high infestation levels without damage (Hepburn and Radloff 1998). Moreover, the same seems to hold true for Africanized honeybee colonies in Brazil, as little to no damage by SHB has been reported in Latin America (Bulacio Cagnolo et al. 2023), despite moderate infestation levels. This supports the notion that quantitative differences in a range of behaviours between honeybee host populations play important roles in governing the impact of SHB (Neumann and Elzen 2004). In invasive SHB populations that infest European-derived honeybee hosts, higher infestation levels may be connected to the amount of damage (Spiewok et al. 2007), but considering the present data, further research in the invasive ranges will be required to reassess this relationship. In particular, there seems to be an apparent need to study in more detail the possible role of quantitative behavioural differences between host populations (e.g. in aggression (Elzen et al. 2001) or preparation for absconding (Neumann et al. 2018)) for the apparent differences in SHB damage (Neumann and Elzen 2004). Data on Africanized honeybees are especially scarce despite their importance in the tropical areas of the SHB invasion front.

High infestations in the native range despite the apparent absence of SHB mass reproduction in managed colonies (Neumann and Elzen 2004) might be due to frequent reproduction in debris of traditional African bark hives and wild colonies (Ouessou et al. 2018). Indeed, there is often ample debris in bark hives and wild nests compared to western hives (PN, personal observations). Since the vast majority of honeybee colonies in Africa is wild (Hepburn and Radloff 1998), it seems as if the importance of SHB reproduction in debris (Spiewok and Neumann 2006) has been underestimated. Indeed, reproduction in debris can be common in Benin and sufficient to explain the SHB infestation levels of local host colonies (Ouessou et al. 2018). Therefore, a better understanding of the modes of SHB reproduction is required to explain local infestation levels (e.g. on combs vs. in debris). Finally, reproduction associated with beekeeping, e.g. in honey houses (Spiewok et al. 2007), or in association with hosts other than *A. mellifera*, e.g. stingless bees (Mutsaers 2006; Neumann et al. 2016; Nacko et al. 2020), may explain the observed SHB infestation levels in the native range, although more research is needed to fully understand the importance of these factors. In any case, the apparent differences in the present dataset compared to earlier comparisons between endemic and invasive ranges of SHB (Spiewok et al. 2007) seem to derive from differences between apiaries within the ranges.

Indeed, apiary-level differences contributed to varying SHB infestation, and this variation might differ between locations. However, such variation was not explained in our data by previous movements of colonies, past management practices applied to colonies against SHB, or the number of colonies in the apiary. This suggests that while long-range migratory beekeeping was an obvious driver of SHB spread in the USA (Neumann and Elzen 2004) and Australia (Spooner-Hart et al. 2017), short-range transport of colonies and the preparation of colonies for transport is unlikely to be a major driver of SHB mass reproduction and therefore local infestation levels. Further, efficient SHB control (Hood 2011; Neumann et al. 2016) would be expected to impact SHB infestations. It may well be that the SHB management had a short-term but not a long-term effect. Additionally, the available control methods, with few exceptions (Neumann and Hoffmann 2008; Muturi et al. 2022), have not yet been tested for their efficacy. This shows the need for greater efforts to enhance SHB management (Schäfer et al. 2019). The lack of a significant correlation between the number of colonies at an apiary (aka olfactory cues) and SHB infestations agrees well with previous observations suggesting that dispersal by adult SHB is probably limited (Cornelissen et al. 2024; Federico et al. 2025). Nevertheless, other apiary factors are likely important in explaining SHB infestation levels, as highlighted by findings such as the significantly higher SHB infestations of shaded host colonies compared to sun-exposed ones in both native and invasive ranges (Akinwande and Neumann 2018; Weinstein et al. 2024). Other possible apiary factors include adult SHB dispersal (Tribe 2000; Spiewok et al. 2008; Cornelissen et al. 2024), human factors (pest management and migratory beekeeping), and random environmental ones (e.g. weather, Cornelissen et al. 2019). Future studies should therefore aim to include a wider spatial scale of apiaries within areas, as well as more detailed variables for environmental factors at apiaries and beekeeping practices. Considering our results, understanding the drivers of the observed variation in SHB infestation levels between apiaries will be important for reducing beetle-induced colony losses in the invasive ranges.

Surprisingly, our results show that SHB infestation levels are lower in Italy than all other studied locations, despite suitable climate and the population being well-established since 2014 (Palmeri et al. 2015). This is most likely due to the rapid implementation of control measures against SHB, such as destroying infested colonies and banning all movement of bees and beekeeping material in the protection zone (Mutinelli 2014). In comparison, no control measures were undertaken in Brazil after SHB was detected in 2015 (Bulacio Cagnolo et al. 2023), which possibly explains the significant difference in infestation levels in these two locations despite the invasions being detected approximately at the same time. Additionally, the climate between these two locations is quite different, with colder temperatures and less rainfall in the Mediterranean climate in Italy compared to the tropical Atlantic climate in Brazil, being more optimal for SHB reproduction (reviewed by Neumann et al. 2016).

While seasonal variation in abiotic factors, i.e. temperature and rainfall, may explain some of the differences in SHB infestation levels between locations, it is unlikely that they explain the full pattern. Many of the locations display similar patterns of colder winter months, and yet, the SHB infestation levels between, for example, RSA and Maryland, are significantly different. However, these environmental factors likely explain temporal variation in infestation levels within populations. We found higher SHB infestation levels during summer than spring in RSA, which could be explained by the colder winter months. Our findings also support previous reports of SHB being present all year round (Lundie 1940) but not reproducing during winter (Neumann et al. 2016). Our results also show that SHB infestation levels were higher in Kenya a few weeks after the rainy season ended as opposed to after an extended dry season, suggesting that pupating SHB may be unable to tolerate extremely low soil moisture for long periods of time, as the dry season had been exceptionally long during our data collection. SHB larvae are not able to pupate in completely dry soil (Cornelissen et al. 2019), and after months of dry season, the top layer of soil may be too dry for the pupae to survive. Additionally, dry seasons also affect the foraging of the honeybee hosts, leading to fewer colony resources as well as possible seasonal migration (Hepburn and Radloff 1998). The confirmed seasonal variation in SHB infestation levels also suggests that shifts in environmental factors caused by climate change may further impact SHB infestations and promote new invasions (Cornelissen et al. 2019).

We found no significant yearly fluctuations in SHB infestation levels in Italy, Brazil, or Alabama, suggesting that these invasive populations are all well-established and no longer show growth or stochasticity typical of new invasive populations (Sakai et al. 2001). This likely differs at the invasion front since most new SHB invasions probably consist of only a few individuals, and it often takes some years for a population to become established in an area (Neumann et al. 2016). Indeed, this seems to hold true in the SHB invasion front in Costa Rica (Villalobos et al. 2025).

In conclusion, our data show that adult SHB infestation levels are not lower in the native range compared to many well-established invasive ranges. The impact of SHB on its honeybee hosts seems to be more related to behavioural differences between host populations (reviewed by Neumann and Elzen 2004) than the sheer number of adult SHB infesting the host. This highlights interesting differences to other host-parasite systems, where negative fitness consequences on hosts tend to increase with parasite load (Hudson et al. 2002). Future studies should aim to better understand variations in host susceptibility as well as SHB aggregation to mitigate the damage caused to apiculture in the invasive ranges of SHB. Our data also suggest that the control measures against SHB in Italy over the last 10 years (reviewed by Federico et al. 2025) have slowed down the growth of the invasive population there. However, as the drivers of parasite-host dynamics are often complex and context-dependent (Sweeny et al. 2021), SHB infestation levels should be monitored repeatedly over time and combined with estimates of reproduction and mortality across a range of environmental variables to further understand and model the spatiotemporal dynamics of SHB populations.

## Supplementary Information

Below is the link to the electronic supplementary material.

ESM 1 (93.4 KB DOCX)

## Data Availability

The raw data of the study are on Dryad repository under 10.5061/dryad.0k6djhbb4.
