# Supplementary material for: Spatiotemporal variation of small hive beetle infestation levels in honeybee host colonies
Source: Apidologie. 2025 Sep 4;56(5):79. doi: 10.1007/s13592-025-01206-8 (PMC12411581; doi:10.1007/s13592-025-01206-8)
Supplement: Supplementary file 1 — (93.4 KB DOCX) [file 13592_2025_1206_MOESM1_ESM.docx]

Apidologie

**Spatiotemporal variation of small hive beetle infestation levels**

**in honeybee host colonies**

Aura Palonen, Anna Papach, Michael N.K. Muturi, Érica Weinstein Teixeira, Geoffrey R. Williams, Rachel Jacobsen, Jay D. Evans, Francisco J. Posada-Florez, Christian W. W. Pirk, H. Michael G. Lattorff, Akinwande K. Lawrence, Murele O. Oluseyi, Robert Spooner-Hart, Clarissa M. House, Giovanni Federico, Giovanni Formato and Peter Neumann

Corresponding author: Aura Palonen; Institute of Bee Health, Vetsuisse Faculty, University of Bern, Bern, Switzerland; aura.palonen@unibe.ch

ELECTRONIC SUPPLEMENTARY MATERIAL

**Table SI.** Location- and apiary-level infestation of small hive beetle, *Aethina tumida* (SHB). Number of screened colonies at each apiary, as well as the apiary and location infestation levels (median, 1^st^ and 3^rd^ quartiles) are shown.

| **Location** | **Apiary** | **N screened colonies** | **Apiary infestation level median [1st;3rd]** | **Location infestation level median [1st;3rd]** |
| --- | --- | --- | --- | --- |
| Italy | IT1 | 20 | 3 [2;6] | 1 [0;4] |
|  | IT2 | 20 | 3 [0;6] |  |
|  | IT3 | 20 | 0 [0;0] |  |
| USA Maryland | MD1 | 10 | 3 [0;6] | 8 [4;19] |
|  | MD2 | 10 | 17 [9;27] |  |
|  | MD3 | 10 | 8 [4;11] |  |
| Brazil | BR1 | 20 | 3 [2;12] | 17 [4;32] |
|  | BR2 | 20 | 19 [8;40] |  |
|  | BR3 | 20 | 29 [17;40] |  |
|  | BR4 | 10 | 18 [10;28] |  |
| USA Alabama | AL1 | 19 | 28 [22;36] | 23 [16;34] |
|  | AL2 | 10 | 20 [12;37] |  |
|  | AL3 | 10 | 13 [6;21] |  |
| Australia | AU1 | 10 | 118 [95;159] | 34 [21;106] |
|  | AU2 | 10 | 23 [19;32] |  |
|  | AU3 | 10 | 25 [12;33] |  |
| Nigeria | NI1 | 10 | 25 [21;39] | 41 [27;54] |
|  | NI2 | 10 | 52 [44;58] |  |
|  | NI3 | 10 | 41 [32;54] |  |
| RSA | RSA1 | 10 | 47 [21;52] | 106 [54;141] |
|  | RSA2 | 10 | 133 [116;162] |  |
|  | RSA3 | 10 | 114 [82;160] |  |
| Kenya | KE1 | 4 | 60 [21;93] | 66 [42;101] |
|  | KE2 | 4 | 89 [54;126] |  |
|  | KE3 | 4 | 65 [48;93] |  |


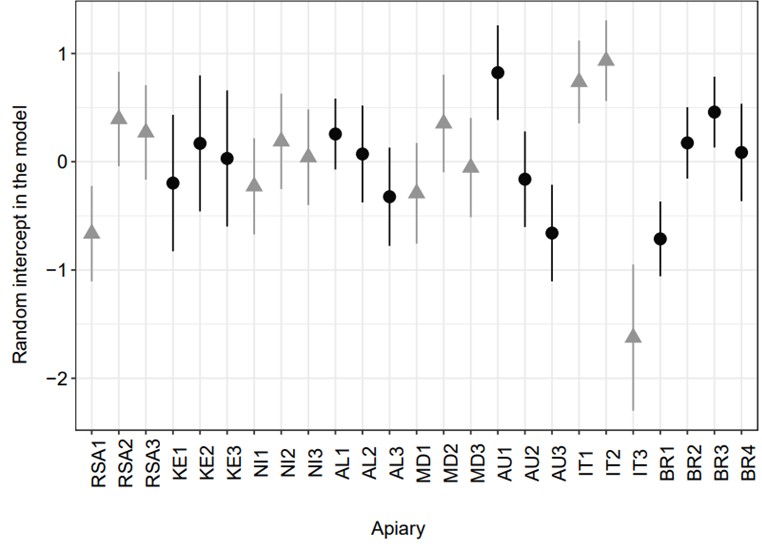


**Figure S1.** Local variation in small hive beetle, *Aethina tumida*, infestations of honeybee, *Apis mellifera*, host colonies at the apiary level estimated by the final generalized linear mixed model. The y- axis represents the random intercepts in the model, and the x-axis represents the apiaries. Abbreviations are: RSA = Republic of South Africa, KE = Kenya, NI = Nigeria, AL = Alabama (USA), MD = Maryland (USA), AU = Australia, IT = Italy and BR = Brazil. Variance between apiaries differs by location, with more even distributions in some locations (e.g. Kenya and Nigeria) compared to others (e.g. Brazil, Italy and Australia).
